# PHOSIDA (phosphorylation site database): management, structural and evolutionary investigation, and prediction of phosphosites

**DOI:** 10.1186/gb-2007-8-11-r250

**Published:** 2007-11-26

**Authors:** Florian Gnad, Shubin Ren, Juergen Cox, Jesper V Olsen, Boris Macek, Mario Oroshi, Matthias Mann

**Affiliations:** 1 Department for Proteomics and Signal Transduction, Max-Planck Institute for Biochemistry, Am Klopferspitz, D-82152 Martinsried, Germany

## Abstract

PHOSIDA , a phosphorylation site database, integrates thousands of high-confidence *in vivo *phosphosites identified by mass spectrometry-based proteomics in various species. For each phosphosite, PHOSIDA lists matching kinase motifs, predicted secondary structures, conservation patterns, and its dynamic regulation upon stimulus. Using support vector machines, PHOSIDA also predicts phosphosites.

## Abstract 2

PHOSIDA, a phosphorylation site database, integrates thousands of phosphosites identified by proteomics in various species.

## Rationale

Protein phosphorylation is a ubiquitous and important post-translational modification, responsible for modulating protein function, localization, interaction and stability [1-4]. High-throughput experimental studies such as our recent large scale analysis of the human phosphoproteome by quantitative mass spectrometry, in which we measured the time courses of more than 6,600 phosphorylation sites in response to growth factor stimulation [5], enable us to study biological systems from a global perspective. Those sites were identified by high resolution mass spectrometry with an estimated false positive rate of less than one percent and constitute an unbiased, in-depth sampling of the *in vivo *phosphoproteome. In addition, PHOSIDA includes large-scale phosphoproteomes from various eukaryotic and prokaryotic organisms, such as *Bacillus subtilis *[6] and *Escherichia coli*, providing information about the evolution of phosphorylation events in the cell.

We developed PHOSIDA to retrieve and analyze phosphosites from large-scale and high-confidence quantitative phosphoproteomics experiments, usually studying the response of biological systems to various stimuli by the integration of time course data. Thus, it is the first phosphosite database to explicitly store quantitative data on the relative level of phosphorylation. PHOSIDA also matches kinase motifs to phosphosites. A challenge in mass spectrometry-based phosphosite mapping is the fact that phosphopeptides are measured, which then need to be mapped to one or more corresponding protein sequences. This problem is addressed in PHOSIDA by a many-to-many mapping between phosphopeptide sequences and protein entries in the sequence database. One of the fundamental strengths of PHOSIDA lies in the high quality of the *in vivo *data contained in the database and in the very large size of its *in vivo *data sets.

In this paper we describe the features and capabilities of PHOSIDA. We also use the analysis tools in PHOSIDA to investigate the structure and evolution of the phosphoproteome from a global point of view. Recent studies have found support for the hypothesis that protein phosphorylation occurs predominantly within regions without regular structure [7,8]. This was also the conclusion of a recent paper describing MitoCheck (mtcPTM) [9], a recently established database containing phosphorylation sites of human and mouse. These authors used known structures and homology modeling to determine the structural constraints of phosphorylation sites. Here we investigate and quantify this observation on a very large *in vivo *dataset. The resulting secondary structure and accessibility information for each phosphosite is available in PHOSIDA.

Although conservation of specific sites is often taken to imply biological importance, relatively little is known about the evolutionary constraints on the phosphoproteome. We investigated these constraints on three levels: conservation of phosphoproteins, regions surrounding the site and the phosphosite itself. Consequently, PHOSIDA provides the evolutionary conservation of each phosphosite at these three levels.

In addition, we took advantage of the large number of *in vivo *phosphosites to create a phosphosite predictor in PHOSIDA. There have been various machine learning approaches to predict phosphorylation sites. For example, the prediction system Netphos [10] is based on neural networks, whereas Scansite uses a profile method to predict phosphorylation events [11]. We use our large-scale studies to construct a phosphorylation site predictor on the basis of a support vector machine (see [12] for an introduction). Support vector machines (SVMs) have been applied to a large variety of fields ranging from internet fraud to topics in molecular biology, such as classification of gene expression profiles, and there has already been one study that applied SVM techniques to predict phosphorylation sites [13]. However, that approach was exclusively based on the primary sequences of around 1,000 phosphorylation sites. Here we construct a predictor based on more than 5,000 high confidence phosphosites. We also show that information about the structure and conservation of phosphorylation sites slightly increases the performance of the predictor.

Furthermore, PHOSIDA can search for motifs of interest in any input sequence. These motifs can be user generated or drawn from already annotated kinase motifs.

## Database management of phosphorylation sites

As mentioned above, PHOSIDA was first developed to facilitate retrieval and analysis of high-confidence phospho-datasets generated in our group. For example, PHOSIDA contains a large number of phosphorylation sites from human cell lines exposed to growth factor stimulation. Protein assignments are based on the IPI database [14], which is cross-referenced with the Swissprot database by PHOSIDA. Entries of both databases that correspond to the same proteins were aligned to derive the exact positions of protein features such as domains, active sites, motifs, and binding sites. Already annotated phosphosites derived from Swissprot are transferred to the IPI sequences in the same way. The aligned regions can be visualized via 'check alignment' buttons. Phosphoproteome data generated by the community will be regularly imported into PHOSIDA in this way rather than by individual import of specific projects. PHOSIDA will be updated with sites identified according to Swissprot every 6 months at the least or as soon as substantial new large-scale studies on phosphorylation are included in Swissprot. In the case of prokaryotic phosphorylation sites, the protein assignment was exclusively based on the TIGR database.

**Figure 1:**
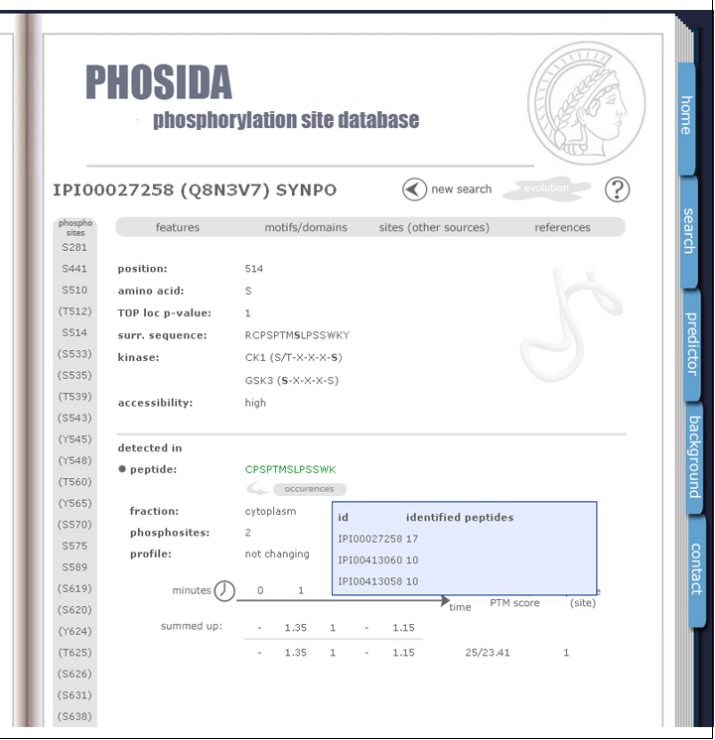
PHOSIDA: phosphorylation site information. For each detected phosphorylation site, the position within the protein sequence along with its surrounding region, maximum assignment localization value, matching kinase motifs, and accessibility is shown. In addition, all detected phosphopeptides that contain the selected phosphosite are displayed along with their corresponding database identification scores, ratios after stimulus, fractions, and occurrences in other proteins.

For each protein, the user is presented with general features such as isoelectric point (pI), molecular weight, sequence, and description at the protein level in addition to all phosphorylation sites that have been identified in our laboratory and those that are extracted from Swissprot. Mass spectrometry identifies phosphopeptides by matching fragmentation spectra to databases and we require 99% confidence for peptide identification to list peptides in PHOSIDA. However, localization of phosphosites within the identified phosphorylated peptides is sometimes ambiguous. We developed a probability score ('localization score') that reflects the chance of each potential phosphorylation site within the peptide to be phosphorylated given its fragmentation spectrum [5]. If the localization probability is lower than 0.995, it is enclosed in round brackets. When users click on any of the displayed phosphosites, the surrounding sequence and matching kinase motifs are shown (Figure 1). Often, several phosphopeptides covering the same phosphosite are measured by mass spectrometry. These peptides are also listed along with their localization probabilities, Mascot scores, and MSQuant scores for each instance. In many cases, for example, the growth factor treatment mentioned above, PHOSIDA contains quantitative and time-resolved data for the relative abundance of each phosphopeptide. Figure 1 shows how the corresponding ratios or clustered time courses are represented. These data are listed separately for peptides as a function of their sequences, degrees of phosphorylation, and further categories, such as experimental design or fraction. When moving the mouse over the 'occurrences' button, protein entries sharing the same phosphopeptide of interest are listed along with the number of unique peptides that have been measured in one experimental assay. Each peptide is color coded according to the protein assignment: if the peptide sequence is marked in green, the selected protein has the maximum number of peptides in comparison to all other proteins that contain the same peptide. If the protein assignment is ambiguous because of another protein with the same number of identified peptides, the peptide is highlighted in blue. Red indicates that other proteins exceed the number of detected peptides in comparison to the selected phosphoprotein. Each feature of PHOSIDA is explained in the help menu, which is accessible via the 'background' menu or via clicking on the 'question mark' button at the page of interest.

## Structural investigation of the phosphoproteome

**Figure 2:**
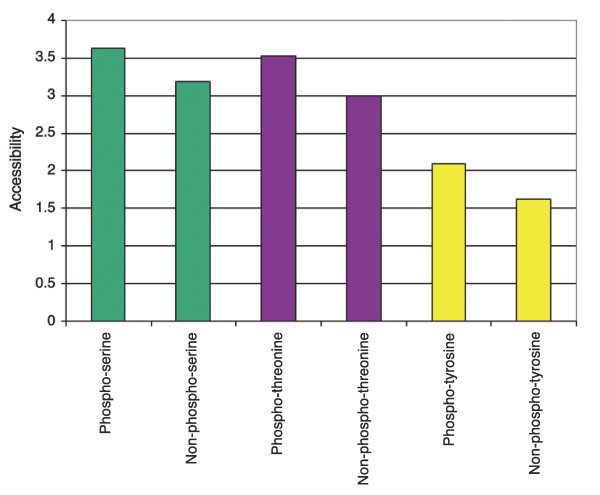
Accessibilities of phosphorylation sites as calculated by SABLE. The relative accessibility prediction assigns a value between 0 (fully buried) and 9 (fully exposed) to each residue. For phosphoserines, phosphothreonines and phosphotyrosines, accessibility is significantly higher than for their non-phosphorylated counterparts in the same proteins.

Previous studies have already shown that phosphorylation sites are mainly located in parts of proteins without regular structure [7,8]. To verify this observation on the basis of our large-scale studies and to enable users to investigate the structural context of each phosphorylation site of interest, we performed large-scale solvent accessibility calculation as well as secondary structure prediction employing the SABLE 2.0 program [15]. As shown in Figure 1, the structural attributes of each phosphorylation site are visualized in PHOSIDA. To determine the overall accessibility at the protein level, we compared 1,044 human phosphoproteins that had an exact match in Swissprot with a set of 998 human random proteins from Swissprot. This was done to avoid bias due to redundant entries in IPI. We find that phosphoproteins as a group have significantly higher accessibilities than a set of randomly selected proteins (*t*-test, σ = 0; Additional data file 1). This means that all residues that occur in phosphoproteins show a higher accessibility on average than all residues in non-phosphorylated proteins. Phosphoproteins, on average, are longer than the average of the database; thus, this effect is not caused by a smaller surface to volume ratio. A global analysis on the human set, which contains 5,849 sites for which the localization was unambiguous (class I sites), showed that the accessibilities of phosphoserine (pS: *t*-test, σ_t _= 2 × 10^-111^; Mann-Whitney test, σ_MW _= 4 × 10^-103^), phosphothreonine (pT: σ_t _= 1 × 10^-21^, σ_MW _= 3 × 10^-21^), and phosphotyrosine (pY: σ_t _= 1 × 10^-4^, σ_MW _= 3 × 10^-4^) are significantly higher than non-phosphorylated serines, threonines or tyrosines (Figure 2). Non-phosphorylated residues were taken from phosphoproteins. Thus, accessibility of phosphoresidues does not only follow from the hydrophilicity of the amino acid but appears to be a requisite for efficient phosphorylation. This finding also correlates with the much higher frequency of pS and pT (80% and 18%, respectively) compared to pY (2%) [5]. Tyrosine is more hydrophobic than serine and threonine and, therefore, tends to be located in less accessible parts of the protein.

**Figure 3:**
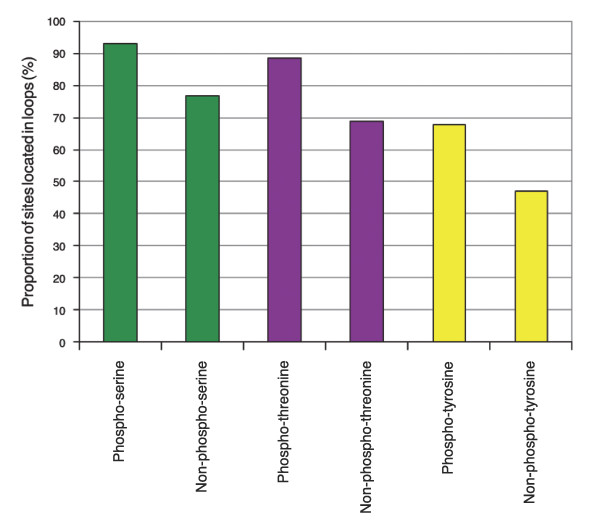
Proportion of phosphorylation sites located in loops and hinges as determined by SABLE. In each case, phosphosites are significantly more frequently located in flexible regions.

The high accessibility of phosphorylation sites suggests that they are largely localized in hinges and loops, since these structural elements are at the protein surface. In fact, this is the case to a striking degree for pS (93.0%) as well as for pT (88.5%). pY (67.3%) is also predominantly found in these regions. Again, this pattern is not caused by the residues' hydrophobicities alone as phosphoresidues have a significantly higher tendency to be located in these regions (χ^2 ^test: *p *= 0 (pS), *p *= 0 (pT), p = 5 × 10^-6 ^(pY); Figure 3). It is well known that loop regions frequently participate in forming binding sites and active sites of enzymes, making them excellent substrates for regulation.

We next wanted to confirm the generality of this observation for phosphoproteins with a solved structure and determined proteins from our human phosphoset that had a structure in the Protein Data Bank [16] and mapped our *in vivo *phosphorylation sites to their three-dimensional coordinates. Secondary structures were assigned by DSSP [17]. DSSP is a program that assigns secondary structures to given three-dimensional coordinates of atoms of proteins. In total, we assigned 26 phosphogroups to 16 structures of different proteins (Additional data file 2). As is apparent from the structures, the phosphogroups are always located in highly accessible parts of the proteins. Furthermore, in all but one case the phosphogroups are found in flexible parts of the structure (hinges or loops). In 12 cases the structure around the phosphosite was so flexible that it had not been determined at all (Additional data file 3).

## Evolutionary conservation of the phosphoproteome

We next wished to integrate another dimension of biological information of the phosphoproteome into PHOSIDA, namely its evolutionary conservation. We determined homologous proteins to all phosphoproteins across 70 species from *E. coli *to mouse via BLASTP [18]. The homology search was performed against protein databases of 53 bacteria, nine archaea, and eight eukaryotes. These databases were retrieved from Swissprot [19] in the case of Archaea and Bacteria. The yeast proteome was downloaded from SGD [20], *Drosophila melanogaster *from FlyBase [21] and the other eukaryotic sequences from IPI. We defined proteins to be homologous when the resulting E-values were lower than 10^-5^. For homologous proteins, we used a bidirectional BLASTP approach to distinguish between paralogs and orthologs [22].

PHOSIDA displays the results of the homology searches using an approximate phylogeny of all investigated species. Taxonomic divisions are displayed on-screen when the cursor is pointed at the phylogenetic tree. If the selected phosphoprotein is not homologous to any protein of a certain organism, that organism is highlighted in red. If the similarity between the sequence of the phosphoprotein and its homologous protein was the significantly best one in both directions, the given organism is highlighted in green. A higher similarity between the sequence of the homologous protein and another protein of the organism of the selected phosphoprotein suggests paralogy, which is indicated in blue.

**Figure 4:**
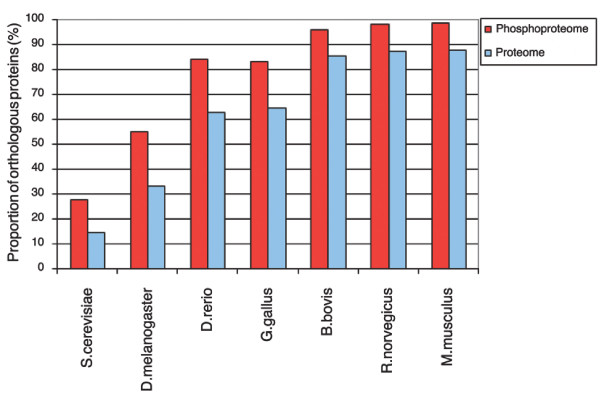
Proportions of phosphoproteins with orthologs. To examine the conservation of phosphoproteins in comparison to the entire human proteome, we aligned two-directionally against the protein sequences of *Saccharomyces cerevisiae*, *D. melanogaster*, *D. rerio*, *Gallus gallus*, *Bos bovis*, *Rattus norvegicus *and *Mus musculus *via BLASTP. Phosphoproteins (red) have a much higher likelihood to have an ortholog than the entire set of human proteins from SwissProt (blue).

We explored the conservation of the identified human phosphoproteome using the dataset of more than 2,200 phosphoproteins from [5]. We investigated phosphoproteins that had an exact sequence match in the Swissprot database (version 48.0). This resulted in a set of 1,044 human phosphoproteins. As shown in Figure 4, phosphorylated proteins have a higher proportion of two-directionally conserved interspecific homologs (χ^2 ^test, *p *= 0) in comparison to the entire human proteome (complete human Swissprot database), presumably reflecting conserved regulatory functions. For example, in the case of *Danio rerio *alignments, we observed that 62.78% of all human proteins had orthologs in comparison to 84.11% of the phospho set.

**Figure 5:**
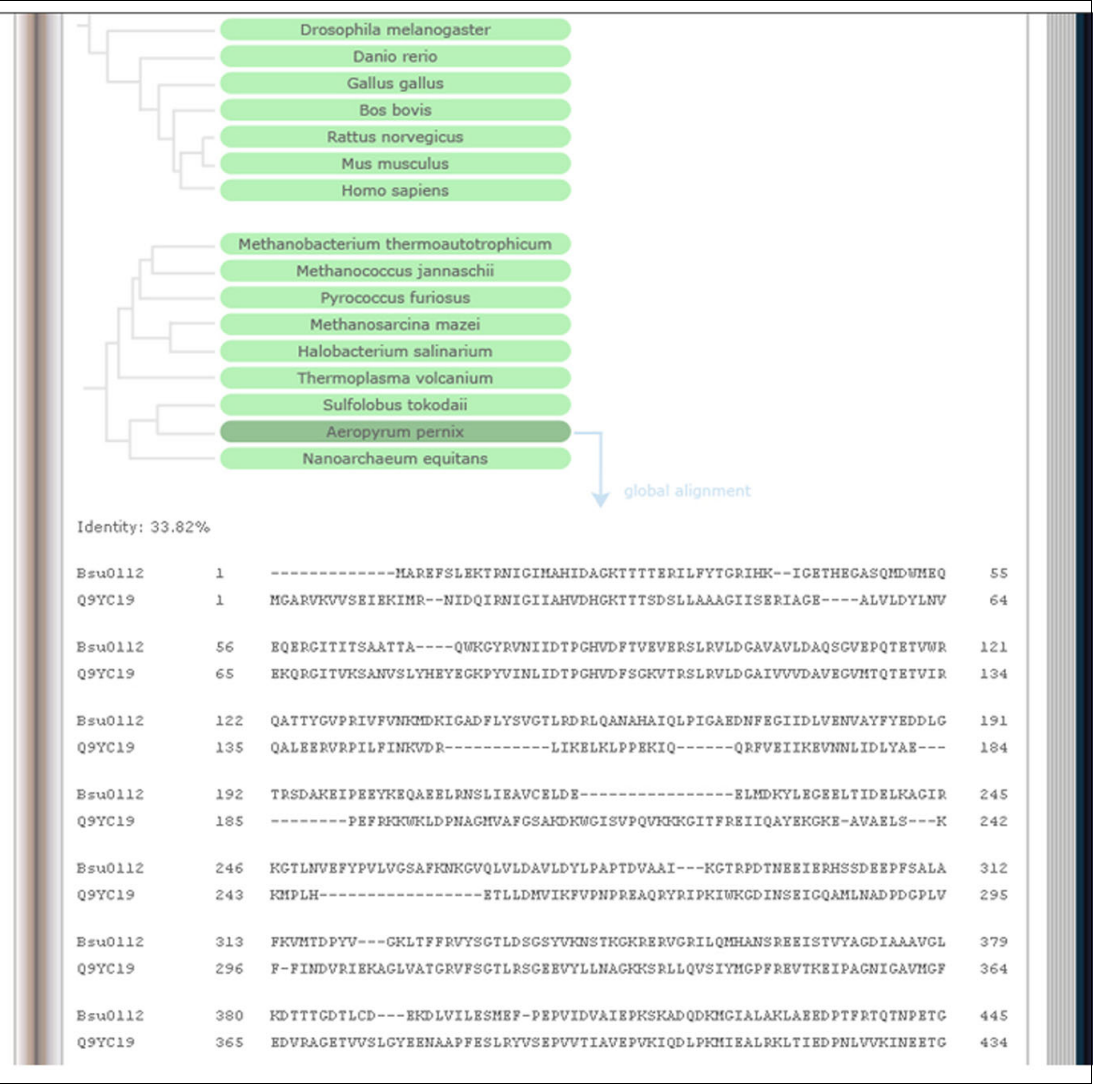
PHOSIDA: evolutionary section. The phylogeny in 70 species is illustrated for each phosphoprotein. The degree of homology is indicated by colors. Red means that the selected phosphoprotein does not show any significant sequence similarity. Blue means that the sequence of the phosphoprotein is significantly similar to a protein of another organism, but only one-directionally according to BLASTP. Green means that the phosphoprotein is probably orthologous to a protein of the chosen organism, since its sequence is significantly similar to the homologous protein in both directions. To enable users to set more stringent criteria for homology relating to the identities of aligned sequences and to check the entire sequence similarity, the global alignments of homologous proteins are also provided.

Additionally, we created global alignments between each phosphoprotein and its corresponding interspecific homolog via the Needleman-Wunsch algorithm [23,24]. Since the length of alignments presents a further criterion for homology besides bi-directional significance via BLAST, users are able to check the global alignments along with the proportions of identities and to estimate the degree of homology by themselves. If users click on any green or blue 'species button', the corresponding global alignment appears at the bottom of the page (Figure 5).

**Figure 6:**
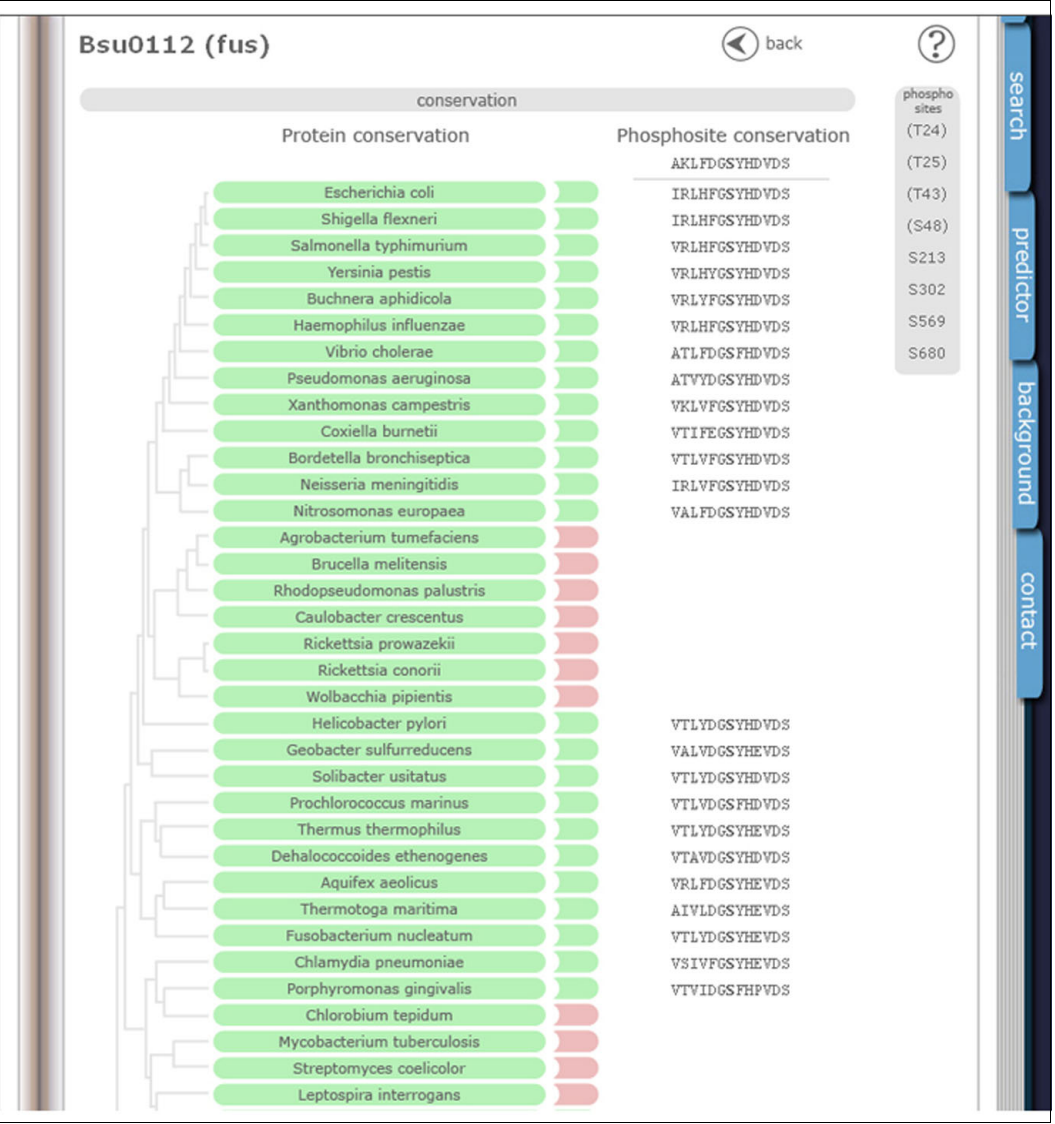
PHOSIDA: evolutionary section. The conservation status of phosphorylation sites within global alignments of homologous proteins is indicated in green or red. Green means that the chosen phosphorylation is conserved. Furthermore, the surrounding aligned sequence is also displayed, to check the conservation of matching kinase motifs.

With global alignments in hand, PHOSIDA directly tests phosphosite and kinase motif conservation. In the evolutionary section of PHOSIDA, all phosphorylation sites that have been measured in our laboratory are listed on the right side. If users click on a phosphorylation site of interest, the conservation status of the selected phosphorylation site is indicated in red or green (Figure 6). Green points to conservation. For conserved phosphosites, the alignment of the surrounding sequence is displayed. Very seldom, sections of the alignments cause gaps in the sequence of the selected short region of the phosphoprotein; in this case these gaps are not displayed. With alignments between the phosphorylation site of interest and protein sequences from 70 organisms, PHOSIDA enables users to check the conservation of each site of each protein of interest. Furthermore, the conservation of matching motifs can immediately be checked as shown in Figure 6. This enables the user to distinguish conserved motifs around the phosphosite from other motifs that also match the phosphosite but are not conserved and may thus be less likely to be functionally important or have appeared only recently in evolution.

On the basis of these global alignments for orthologous phosphoproteins, we found that regions containing phosphorylation sites showed lower conservation than the average conservation of the entire protein. As seen in Additional data file 4, the average identity in the 40 amino acid window surrounding the aligned phosphorylation sites is lower for each eukaryotic species compared to the entire protein identity. This effect is most pronounced for serine and threonine due to their almost exclusive location in fast evolving loop and hinge regions.

These data suggest that the surrounding sequence regions may diverge to such an extent that the structural effect (fast sequence evolution) could compete with the constraining pressure of function (slow sequence evolution). In order to correctly assess the degree of conservation of phosphosites, it is therefore important to take the structural effect - fast evolution of loop regions - into account. We did this by choosing only sites located in loop regions for the comparison set, which should isolate the functional, evolutionary constraints on the phosphosite itself.

**Figure 7:**
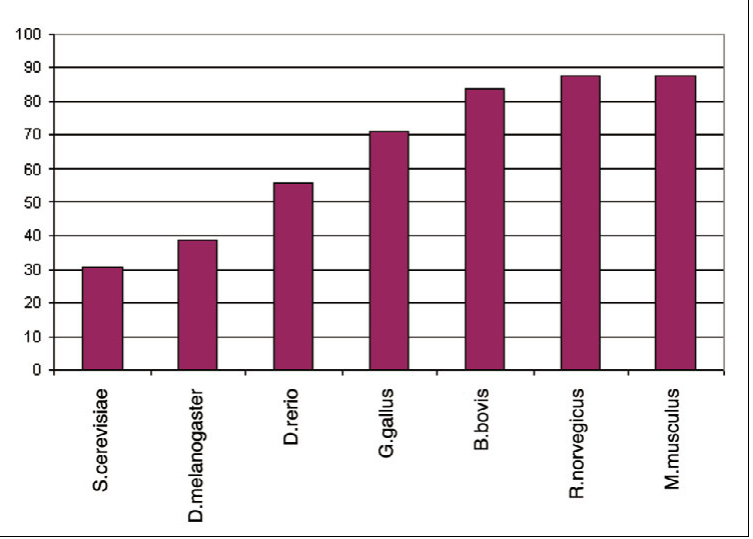
Percentage sequence identity of phosphoproteins with orthologs.

**Figure 8:**
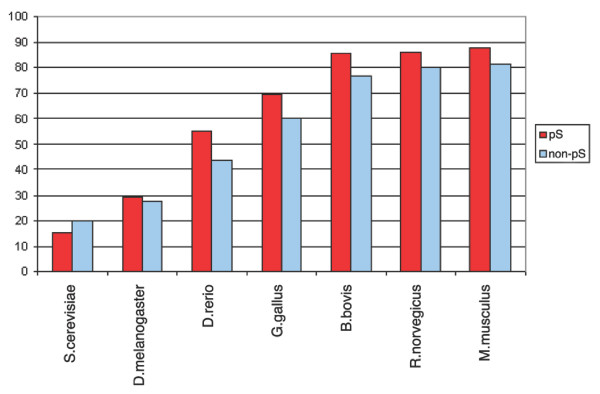
Conservation of phosphoserines (red) compared to non-phosphoserines (blue) in phosphoproteins. Phosphoserines are significantly more conserved except in yeast.

**Figure 9:**
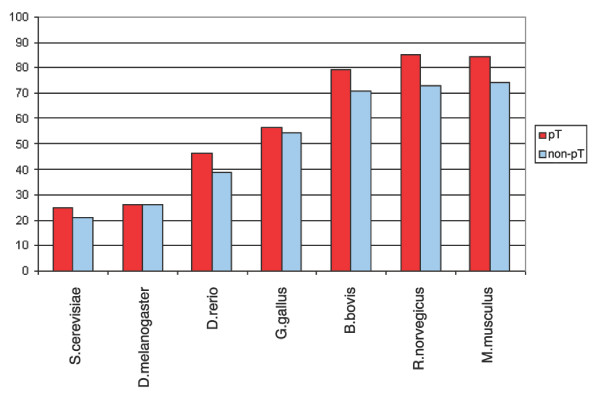
Conservation of phosphothreonines (red) compared to non-phosphothreonines (blue). Phosphothreonines are significantly more conserved within mammals.

**Figure 10:**
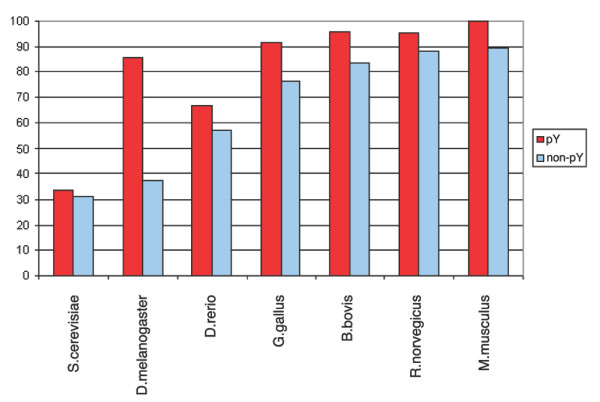
Conservation of phosphotyrosines (red) compared to non-phosphotyrosines (blue). Tyrosine is very highly conserved in mammals in both forms. In more distantly related species the numbers are small and differences are not statistically significant.

The overall conservation of phosphorylation sites in orthologous eukaryotic proteins, based on the Needlemann-Wunsch alignments, is shown in Figures 7, 8, 9, 10. The average amino acid identity for all phosphoproteins with orthologs ranges from greater than 80% in mammals to about 25% in yeast (Figure 7). Figure 8 compares the conservation of phospho-serines that occur in loops with all non-phosphoserines that occur in loops in the same proteins. In all vertebrates, phosphoserine is significantly more conserved than serine (*p *= 0). In *Drosophila *the effect is still observable, but is not significant (*p *= 0.33). In yeast this is not the case. However, because the sequence identity is already relatively high, the absolute values of phosphoserine conservation are not much higher than those of other serines. For example, compared to mice, 87.77 % of the phosphosites are conserved in orthologous proteins, but 81.16% of all serines in loop regions of phosphoproteins are also conserved. Threonine yields a similar result to serine, but this amino acid is generally less conserved than serine (Figure 9). Tyrosine tends to occur in more conserved regions of the protein as mentioned above. Therefore, conservation of all tyrosines in mouse is very high at 89.3% (Figure 10). However, the higher conservation of phosphorylated tyrosines is still evident, but is not significant due to their low number.

**Figure 11:**
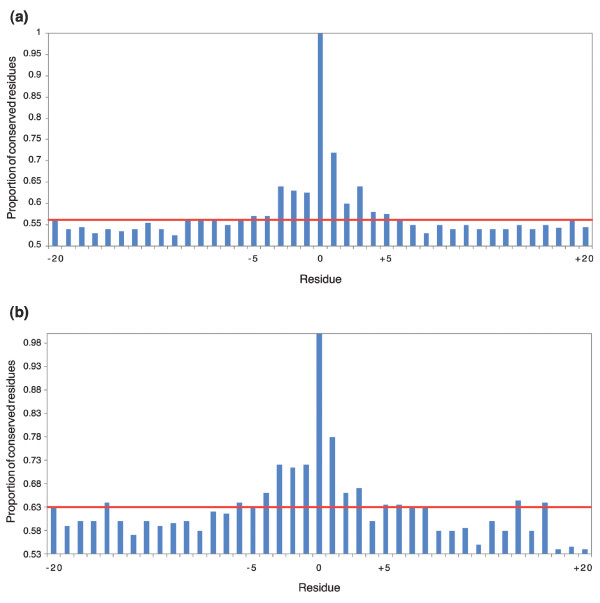
Conservation of phosphorylation motifs. Bars represent the proportion of identical residues in zebrafish orthologs of human phosphoproteins. The red line is the average identity in the region -20 to +20 amino acids surrounding the phosphosite. For both **(a) **serine and **(b) **threonine, about five amino acids in each direction show elevated sequence identity.

What do these findings mean for the conservation of phosphorylation motifs? We plotted the conservation of amino acids amino- and carboxy-terminal to the phosphorylation site for the three phosphorylation sites and for all species. As a typical example, Figure 11 shows the case of serine and threonine in zebrafish (*D. rerio*). The figure reveals a symmetric region immediately adjacent to the phosphosite, in which conservation is higher than in the surrounding region. The length of this region is about -5 to +5 amino acids for both serine and threonine and agrees well with the size of published phosphorylation motifs. Thus, in the evolutionary section of PHOSIDA, the surrounding region of -6 to +6 amino acids is shown, in order to check the conservation of matching motifs. For phosphotyrosine the picture was less clear, perhaps because of the limited number of sites in the data set.

## Prediction of phosphorylation sites using support vector machines

**Figure 12:**
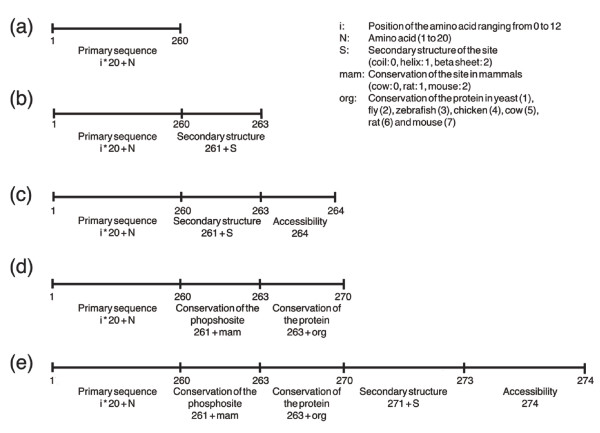
Feature transformation of phosphorylation sites for *in silico *prediction. The surrounding sequence of a phosphorylation site comprises 260 dimensions. Each dimension is defined by the position within the surrounding region and the amino acid type. The possible values in each dimension are 0 and 1. **(a) **Primary sequence **(b) **Extends set a by three dimensions, which include information about the predicted secondary structure of the phosphorylation site. **(c) **Extends set b by one dimension that contains the predicted accessibility. **(d) **Extends set a by three dimensions that reflect the conservation of the phosphosite in mammals and seven additional dimensions that describe the protein conservation in yeast, fly, zebrafish, chicken, cow, rat and mouse. **(e) **Combines set c and set d.

We then used the results of this large-scale study to construct a phosphorylation site predictor on the basis of a SVM. As shown above, phosphoserines, phosphothreonines and phosphotyrosines show the same general patterns relating to protein structure and conservation, but each to a different extent. Therefore, we applied the machine learning approach separately to the 4,731 pS, 664 pT and 107 pY sites. To create a negative set of the same size, we randomly chose sites from human proteins that were not present in the phosphoset. The positive and negative datasets were split into a training set (90%) and a test set (10%). SVMs attempt to partition true from false sites by separating them in a high dimensional vector space with the help of hyperplanes and kernel functions. A few sites out of the negative set may turn out to be phosphorylation sites in future experiments. This problem was addressed by optimizing the 'C parameter' of the SVM, which controls the softness of the margin. We optimized the parameters C and σ by varying them from 2^-10 ^to 2^10 ^in multiplicative steps of two and chose the best combination of both parameters out of the 21 × 21 possibilities. The optimization was based on a five-fold cross validation on the training set. To determine the importance of each feature in the accuracy of phosphosite prediction, we created various sets, which contain different information for each phosphosite (Figure 12): set a, the primary sequence comprising the site and its 12 surrounding residues; set b, the surrounding primary sequence and the predicted secondary structure of the site; set c, the surrounding primary sequence and the predicted accessibility in addition to the secondary structure of the site; set d, the surrounding primary sequence, the conservation of the phosphosite in mammals and the protein conservation in yeast, fly, zebrafish, chicken, cow, rat and mouse; and set e, the surrounding primary sequence, the accessibility of the phosphosite and secondary structure as well as its conservation in mammals, and the protein conservation. This resulted in 260 to 274 dimensions that represent the features of each phosphosite.

We investigated several common kernel functions and found that the radial basis function (RBF) turned out to be the most powerful compared to linear, polynomial and sigmoid Kernel functions. We optimized parameters C and σ, the width of the Gaussians used as the RBFs, and trained the optimal model for each set of each phosphor amino acid separately (Additional data file 5).

We found that the accuracy of the prediction based on the primary sequence was already very high: in the case of phosphoserines, 89.85% were predicted correctly in the test set as were 74.24% of the phosphothreonines (Additional data file 6). The accuracy of the prediction increased to 90.17% for pS and 77.27% for pT by adding structural information (sets b and c). For serines, the accessibility was slightly more important than the secondary structure information, whereas for threonines, the opposite was the case. The additional dimensions reflecting the conservation of the site and of the entire protein (set d) increased the accuracy to 90.70% (pS) and 81.06% (pT). By combining structural and evolutionary information (set e), we found that 91.75% in the serine set and 81.06% in the threonine set were predicted correctly. The accuracy of the prediction of phosphotyrosines increased from 66.67% to 76.19% when including the structural and conservational information. However, that increase is not significant due to the fact that there were only around 100 phosphotyrosines sites.

**Figure 13:**
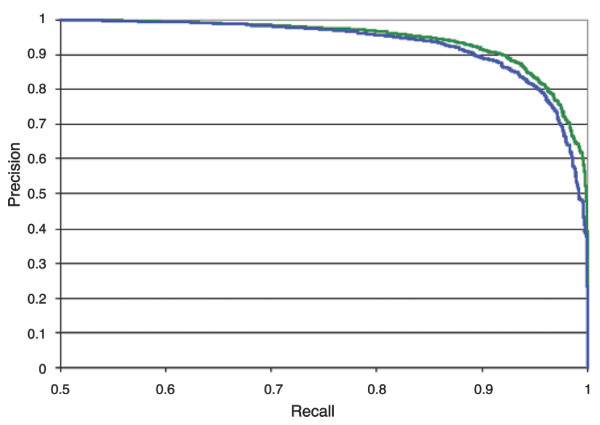
Precision-recall curve for phosphoserines. The two lines present the tradeoff between false positives and false negatives without (blue) and with (green) inclusion of structural and evolutionary constraints.

The recall reflects the proportion of true positives to the sum of true positives and false negatives, whereas the precision describes the number of true positives out of all predicted positives. As outlined in Figure 13, the precision-recall curve of set e is slightly better than that of set a, indicating that the inclusion of evolutionary and structural information increased the recall and precision of the prediction to a minor degree.

PHOSIDA includes the prediction of phosphorylated serines and threonines on any input sequence on the basis of the SVM, which was trained on the basis of raw sequences. Users can set a certain cutoff directly on the precision-recall-curve for the prediction. Sites that are predicted to be phosphorylated are automatically matched to annotated kinase motifs.

In addition to the prediction, we also integrated a simple tool that searches for matching kinase motifs on any sequence of interest. Alternatively, users can define their own motif and derive matching sites of the given sequence.

## Outlook and conclusion

The concept of a phosphorylation site database is, of course, not a novel one. PhosphoSite [25] and Phospho.ELM [26] are already comprehensive databases that contain phosphorylation sites from different projects. The aim of PHOSIDA is to include very high quality input data as well as quantitative information such as regulation after stimuli. Additionally, we take into account structures and evolutionary data across a variety of species, in order to integrate biological context into the database and to quantify constraints of phosphorylation on a proteome-wide scale. Thus, PHOSIDA provides a rich environment to the biologist wishing to analyze phosphorylation events of proteins of interest.

Our analysis of a large and unbiased set of *in vivo *phosphorylation sites in human cells shows that phosphorylation events are not distributed along the whole protein structure but are instead constrained to sites of high accessibility and structural flexibility. Particularly in the case of serine and threonine, phosphorylation is almost completely restricted to loops and hinges. Tyrosine is found to some degree in regular secondary structure elements but phosphotyrosines are very likely to be in flexible regions as well. Mechanistically, localization of phosphorylation in flexible regions of the protein is advantageous as it provides access for the kinase to substrate, which needs to be positioned into the active site. Furthermore, functional consequences of the phosphorylation in many cases also depend on the flexibility of the phosphorylated sequence, such as when loops are repositioned after phosphorylation or when the phosphorylated loop participates in a protein-protein interaction. However, it is important to emphasize that the structural analysis was based on predictive methods rather than experimental data. Nevertheless, it stands to reason that the large size of the dataset should compensate for statistical errors caused by the prediction algorithm. Furthermore, as mentioned above, the Mitocheck database (mtcPTM) [9] also came to similar conclusions relating to structural constraints of phosphosites. This is gratifying because those authors used a different set of phosphorylation sites (gathered in the European Union consortium MitoCheck) and different methods to determine preferential phosphorylation on different secondary structure elements (homology modeling). The authors also noted that phosphorylation sites can accumulate at the flanks of structured domains and, in some cases, on buried residues. Interestingly, phosphorylation of these sites could destabilize part of the protein structure and, for example, allow or disallow protein-protein interactions [9].

The concordant results on structural constraints of phosphorylation sites between the MitoCheck study and this study also implicitly validate our use of the SABLE prediction tool for secondary structure and solvent accessibility prediction in this context. Here we have used these predictions to extend the feature space used in phosphorylation site prediction.

The analysis of the evolutionary sections of PHOSIDA shows that the number of orthologs of the human phosphoproteome is much higher than that of the entire human proteome, at least when analyzing the phosphoproteins identified by Olsen *et al*. [5]. This probably reflects important and conserved functional roles of proteins with this post-translational modification. As a consequence of the location of phosphorylation sites in loops and hinges, the sequence regions around phosphorylation sites evolve faster than the rest of the protein. Practically, this leads to difficulties in correctly aligning phosphosites in orthologous proteins, which can be overcome by using a combination of fast, word-based algorithms (BLASTP) to find candidates and exhaustive algorithms to properly align phosphorylation sites (Needle).

Our analysis on the global alignments of orthologs in eukaryotes shows that phosphorylation sites are more conserved than non-phosphosites of the same proteins. However, for any given site the sequence identity is already very high, for example, more than 70% for serine and threonine in mammals. For tyrosine, conservation is even higher. Therefore, the mere conservation of a phosphorylation site in mammals or in vertebrates does not necessarily indicate high selection pressure. We found that a region of about five amino acids around the phosphorylation site is more conserved than the surrounding sequence context.

Furthermore, we integrated a tool that matches input sequences with annotated kinase motifs or motifs that are defined by users. In addition, we constructed a SVM-based prediction algorithm for phosphorylation. Training of the SVM on our large-scale dataset led to excellent prediction accuracy. We also showed that the inclusion of structural and evolutionary constraints on the phosphoproteome could slightly increase the performance of the predictor.

The PHOSIDA phosphorylation site predictor makes it possible to find putative novel phosphorylation sites that have not (yet) been experimentally identified. While experimental data, especially quantitative data, are the 'gold standard', predicting novel phosphosites and matching kinase motifs on proteins of interest should be valuable for the design of biological experiments or for predicting a protein's role in a pathway [27]. Furthermore, once predictors are trained, these prediction methods are basically 'free'. We provide an interactive method for setting a desired level of precision and recall. For example, for mutagenesis experiments one may want to set the precision very high, and for rationalizing the function of a protein in a pathway one may want to set it relatively low. Thus, in the absence of experimental data, the prediction of novel phosphosites can be taken as the first method of an experimental design uncovering functionality of any protein of interest and elucidating its involvement in certain signaling cascades.

The integration process and analysis pipeline have been automated, so that structural and conservation data for phosphorylation sites from prospective studies can readily be incorporated into PHOSIDA. As new phosphorylation data are integrated to PHOSIDA our SVM will also be updated, leading to increasingly accurate predictions.

Upcoming projects will investigate the phosphoproteomes of prokaryotes, such as *E. coli *and *Lactococcus lactis*, and the dynamics of phosphorylation after various stimuli in *B. subtilis *and in eukaryotes such as *D. melanogaster*, mouse, and human.

## Abbreviations

pS, phosphoserine; pT, phosphothreonine; pY, phosphotyrosine; RBF, radial basis function; SVM, support vector machine.

## Authors' contributions

FG designed and implemented Phosida, performed the analyses and interpreted the bioinformatic findings. SR implemented the support vector machine, JC helped in the design of the support vector machine, JVO and BM contributed to the interpretation of the experimental phosphorylation results, MO contributed to the implementation of Phosida and MM supervised the project and helped write the manuscript. All authors read and approved the final manuscript.

## Additional data files

The following additional data are available with the online version of the paper. Additional data file 1 is a figure showing the accessibilities of phosphorylation sites as calculated by SABLE. Additional data file 2 is a figure showing Protein Data Bank structures of phosphoproteins. Additional data file 3 is a table listing phosphorylation sites located in parts of phosphoproteins that are too flexible for structure determination. Additional data file 4 is a figure that illustrates the conservation of the region surrounding the phosphosite (-20 to +20 amino acids). Additional data file 5 is a table listing the optimal parameters for the SVM prediction. Additional data file 6 is a table listing the prediction accuracies of the SVM approach.

## Supplementary Material

Additional data file 1 The relative accessibility prediction assigns a value between 0 (fully buried) and 9 (fully exposed) to each residue. For phosphoserines, phosphothreonines and phosphotyrosines, accessibility is significantly higher than for their non-phosphorylated counterparts in the same proteins. The overall accessibility of phosphoproteins is also significantly higher than for a random set of around 1,000 human proteins in Swissprot. Click here for file

Additional data file 2 We visualized determined structures of phosphoproteins via Molsoft ICM Browser Pro (version 3.4-8f). Click here for file

Additional data file 3 Phosphorylation sites located in parts of phosphoproteins that are too flexible for structure determination. Click here for file

Additional data file 4 **(a) **Conservation of phosphoserine surrounding sequences (green) in comparison to the average conservation of phosphoproteins (red). **(b) **Conservation of phosphothreonine surrounding sequences (claret-red). **(c) **Conservation of phosphotyrosine surrounding sequences (yellow). Regions around phosphosites are significantly less likely to be conserved than phosphoproteins on average. Click here for file

Additional data file 5 Optimal parameters for the SVM prediction. Click here for file

Additional data file 6 Prediction accuracies of the SVM approach. Click here for file
