# Supplementary figures and images for: PHOSIDA (phosphorylation site database): management, structural and evolutionary investigation, and prediction of phosphosites
Source: Genome Biol. 2007 Nov 26;8(11):R250. doi: 10.1186/gb-2007-8-11-r250 (PMC2258193; doi:10.1186/gb-2007-8-11-r250)

accessibility

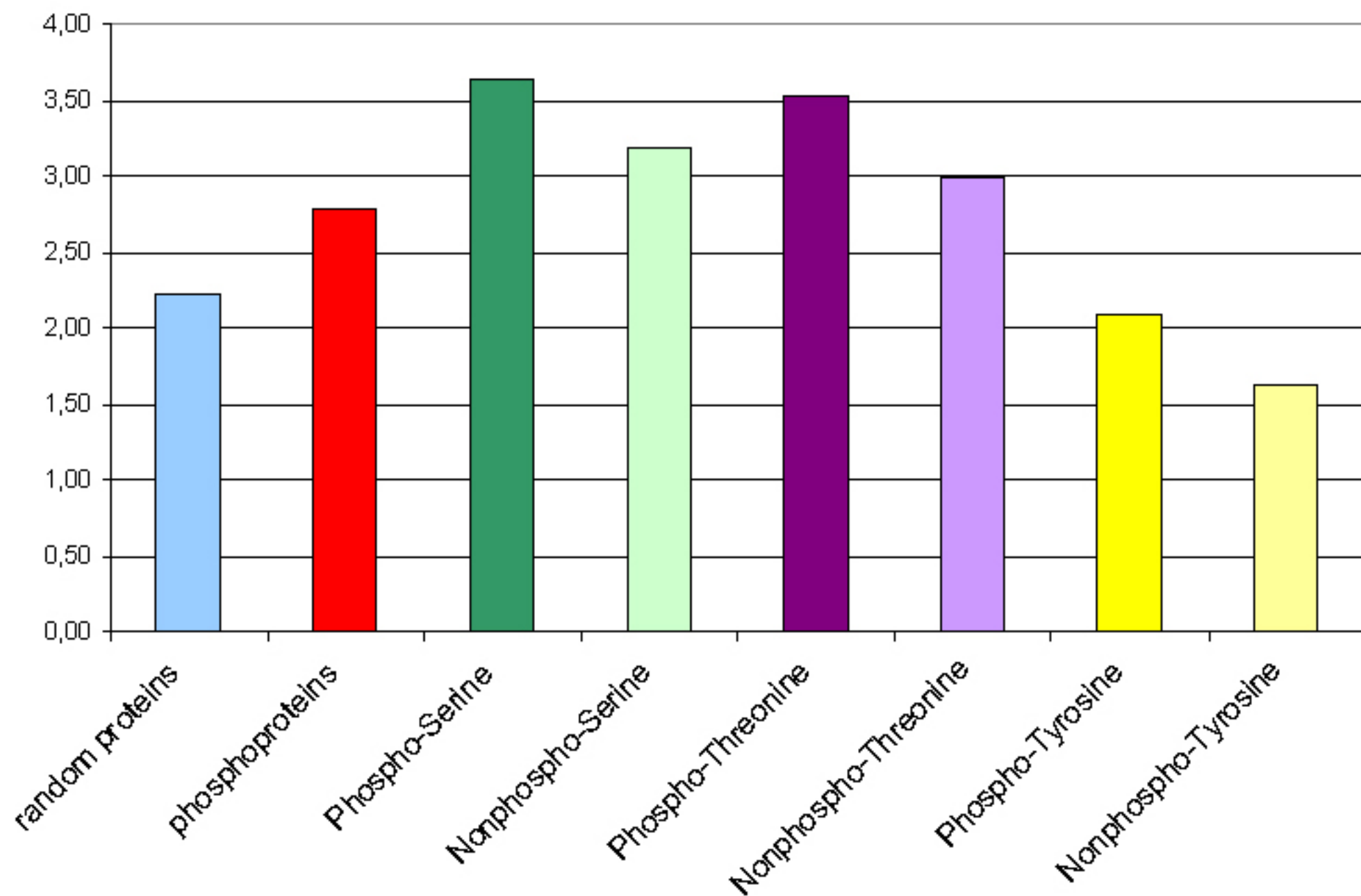

Supplement: Additional data file 1 — The relative accessibility prediction assigns a value between 0 (fully buried) and 9 (fully exposed) to each residue. For phosphoserines, phosphothreonines and phosphotyrosines, accessibility is significantly higher than for their non-phosphorylated counterparts in the same proteins. The overall accessibility of phosphoproteins is also significantly higher than for a random set of around 1,000 human proteins in Swissprot. [file gb-2007-8-11-r250-S1.pdf]

**a**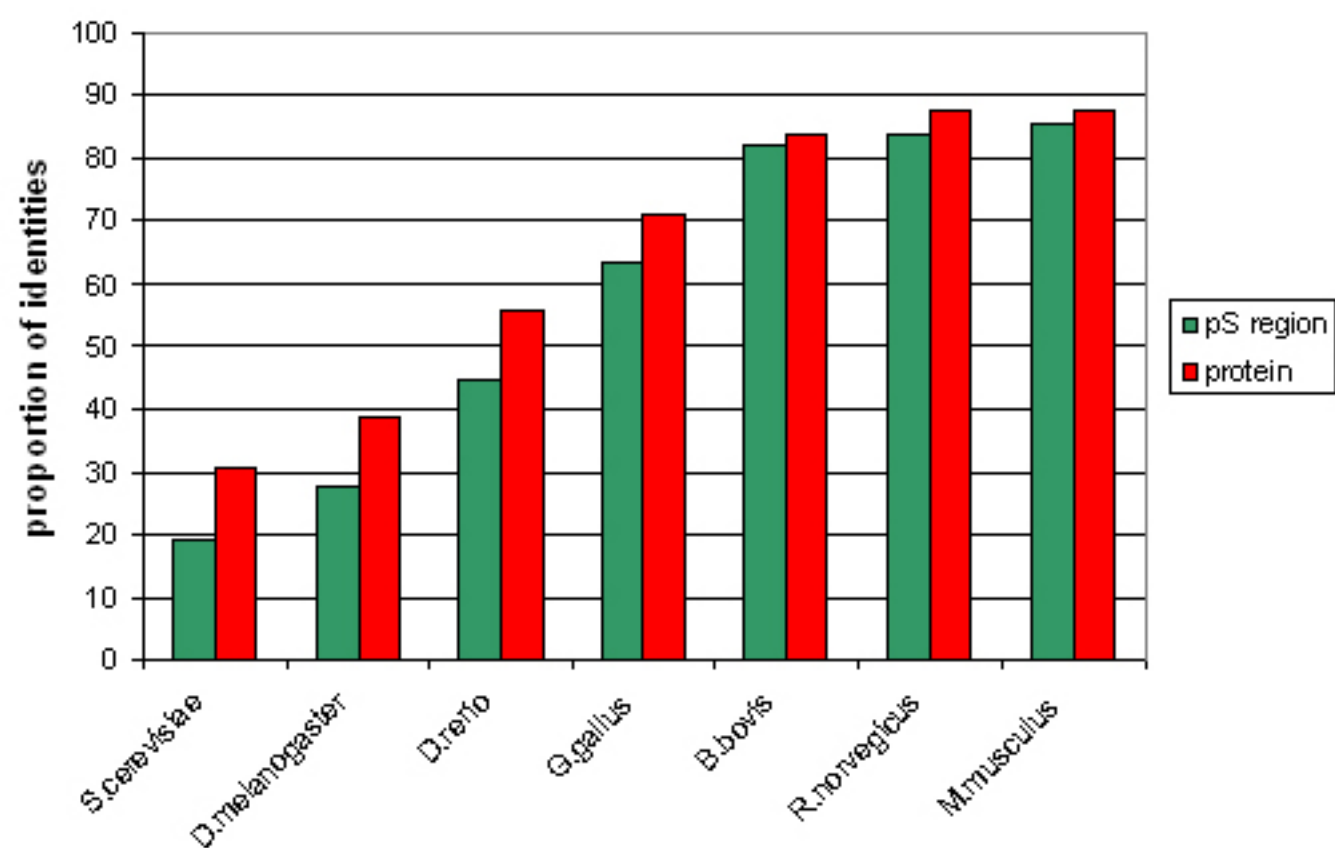**b**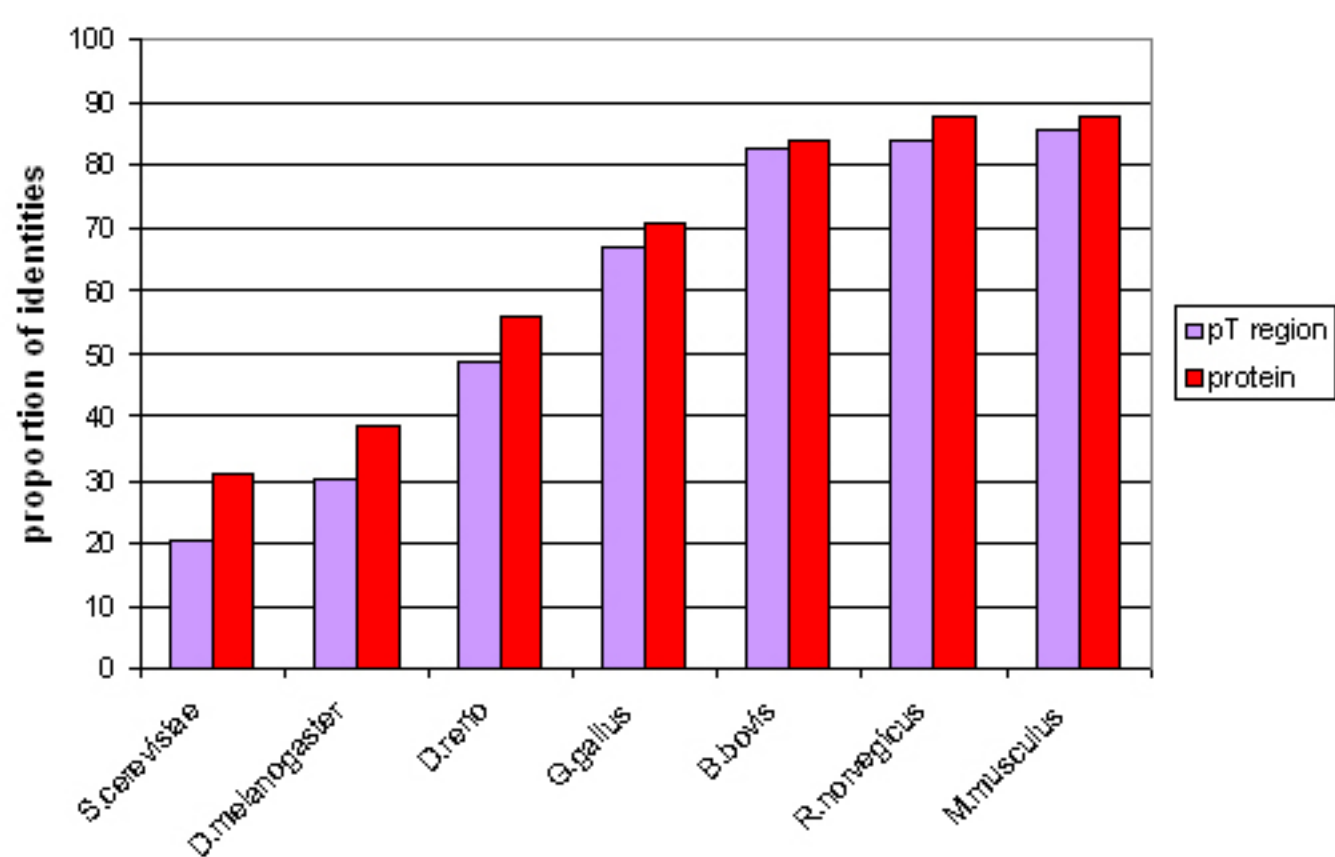**c**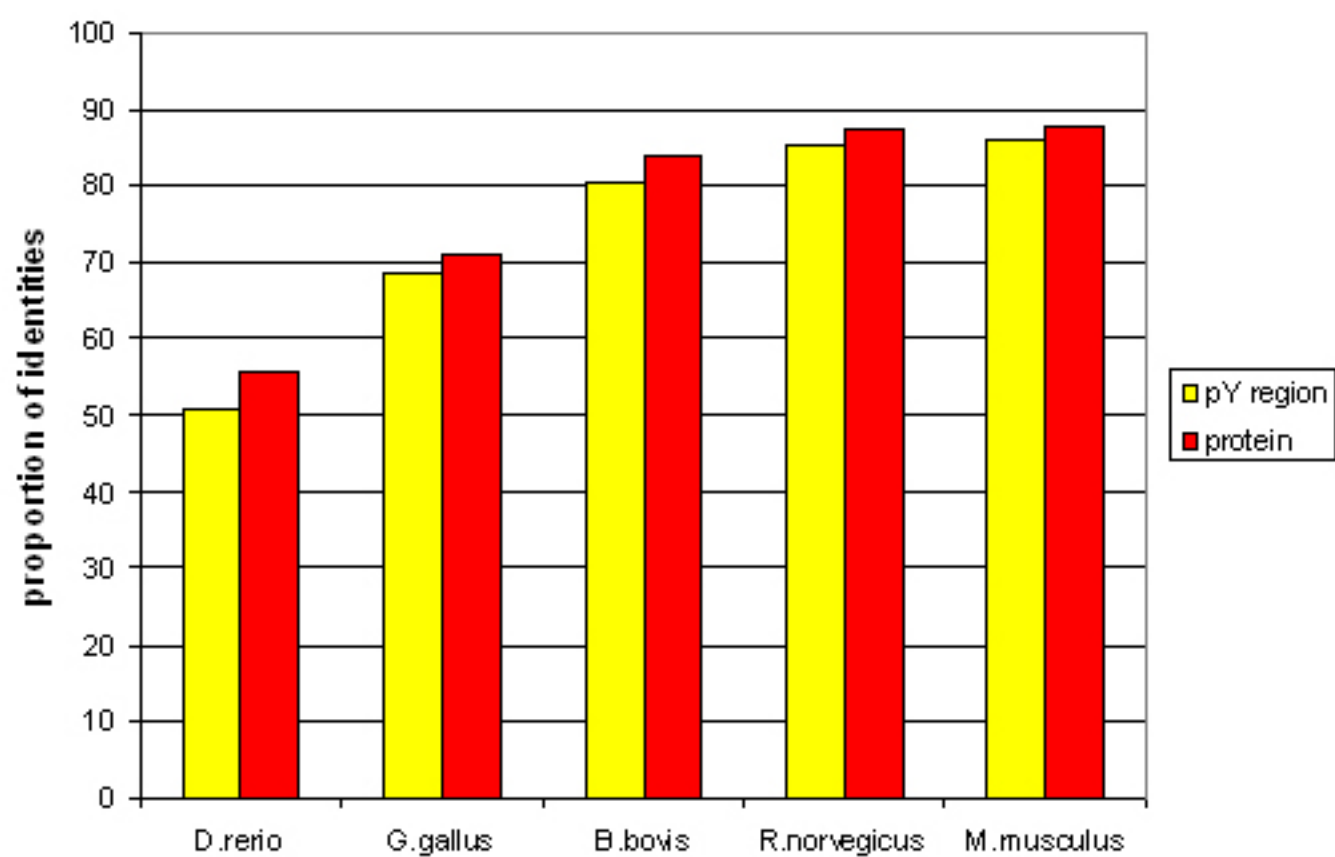

Supplement: Additional data file 4 — (a) Conservation of phosphoserine surrounding sequences (green) in comparison to the average conservation of phosphoproteins (red). (b) Conservation of phosphothreonine surrounding sequences (claret-red). (c) Conservation of phosphotyrosine surrounding sequences (yellow). Regions around phosphosites are significantly less likely to be conserved than phosphoproteins on average. [file gb-2007-8-11-r250-S4.pdf]
