# Supplementary material for: PHOSIDA (phosphorylation site database): management, structural and evolutionary investigation, and prediction of phosphosites
Source: Genome Biol. 2007 Nov 26;8(11):R250. doi: 10.1186/gb-2007-8-11-r250 (PMC2258193; doi:10.1186/gb-2007-8-11-r250)
Supplement: Additional data file 2 — We visualized determined structures of phosphoproteins via Molsoft ICM Browser Pro (version 3.4-8f). [file gb-2007-8-11-r250-S2.pdf]

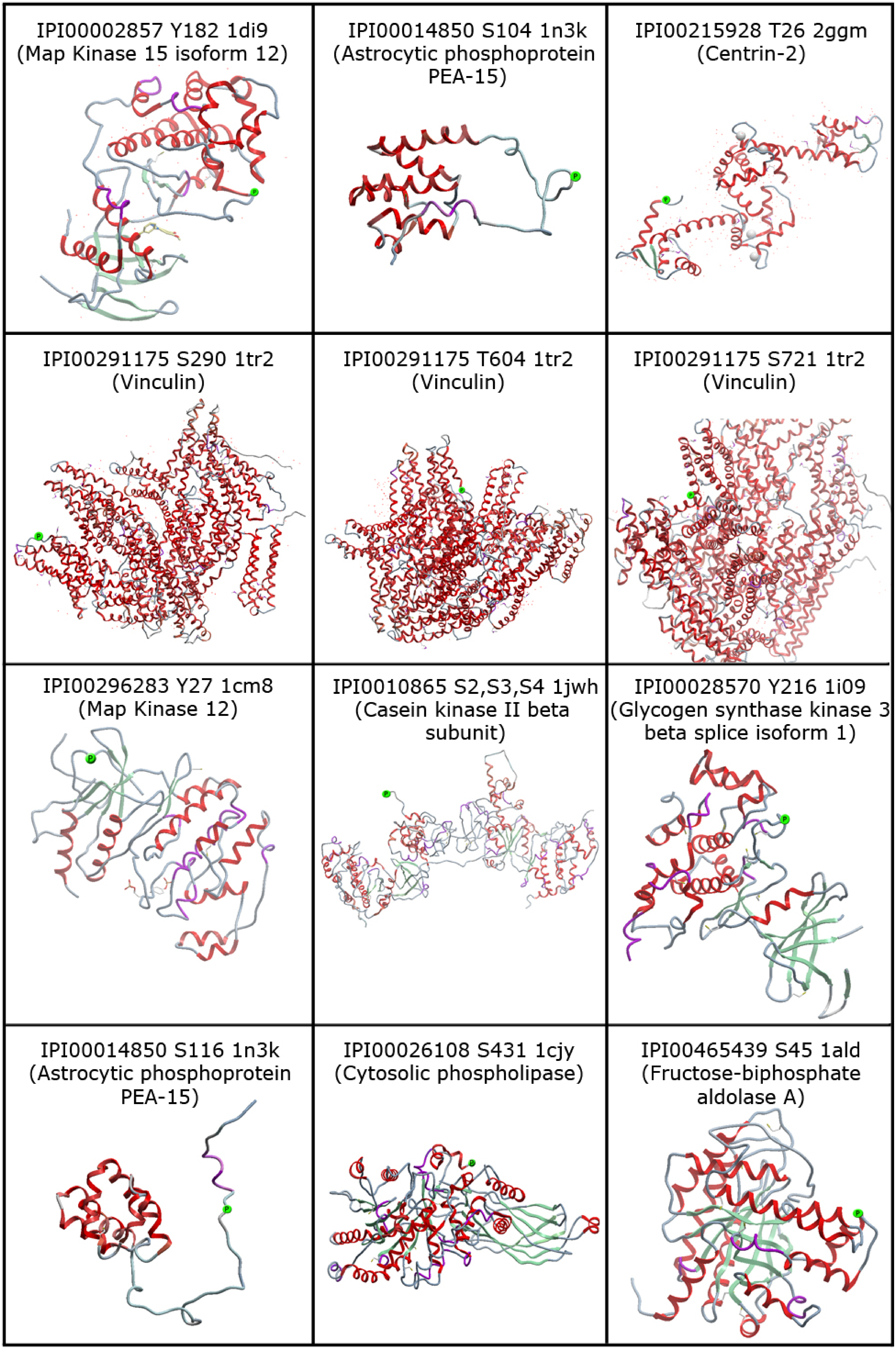
IPI00002857 Y182 1di9
(Map Kinase 15 isoform 12)
IPI00014850 S104 1n3k
(Astrocytic phosphoprotein PEA-15)
IPI00215928 T26 2ggm
(Centrin-2)
IPI00291175 S290 1tr2
(Vinculin)
IPI00291175 T604 1tr2
(Vinculin)
IPI00291175 S721 1tr2
(Vinculin)
IPI00296283 Y27 1cm8
(Map Kinase 12)
IPI0010865 S2,S3,S4 1jwh
(Casein kinase II beta subunit)
IPI00028570 Y216 1i09
(Glycogen synthase kinase 3 beta splice isoform 1)
IPI00014850 S116 1n3k
(Astrocytic phosphoprotein PEA-15)
IPI00026108 S431 1cjy
(Cytosolic phospholipase)
IPI00465439 S45 1ald
(Fructose-biphosphate aldolase A)
